# Enhancing the implantation of mechanical circulatory support devices using computational simulations

**DOI:** 10.3389/fbioe.2024.1279268

**Published:** 2024-04-25

**Authors:** Gabriela Lopez-Santana, Alessandro De Rosis, Stuart Grant, Rajamiyer Venkateswaran, Amir Keshmiri

**Affiliations:** ^1^ School of Engineering, The University of Manchester, Manchester, United Kingdom; ^2^ Department of Cardiothoracic Transplantation and Mechanical Circulatory Support, Wythenshawe Hospital, Manchester, United Kingdom; ^3^ Manchester University NHS Foundation Trust, Manchester Academic Health Science Centre, Manchester, United Kingdom; ^4^ Division of Cardiovascular Sciences, School of Medical Sciences, Faculty of Biology, Medicine and Health, The University of Manchester, Manchester, United Kingdom

**Keywords:** HeartMate, left ventricular assist device, heart failure, computational fluid dynamics, turbulent flow, aortic flow, outflow graft, surgical optimisation

## Abstract

**Introduction:** Patients with end-stage heart failure (HF) may need mechanical circulatory support such as a left ventricular assist device (LVAD). However, there are a range of complications associated with LVAD including aortic regurgitation (AR) and thrombus formation. This study assesses whether the risk of developing aortic conditions can be minimised by optimising LVAD implantation technique.

**Methods:** In this work, we evaluate the aortic flow patterns produced under different geometrical parameters for the anastomosis of the outflow graft (OG) to the aorta using computational fluid dynamics (CFD). A three-dimensional aortic model is created and the HeartMate III OG positioning is simulated by modifying (i) the distance from the anatomic ventriculo-arterial junction (AVJ) to the OG, (ii) the cardinal position around the aorta, and (iii) the angle between the aorta and the OG. The continuous LVAD flow and the remnant native cardiac cycle are used as inlet boundaries and the three-element Windkessel model is applied at the pressure outlets.

**Results:** The analysis quantifies the impact of OG positioning on different haemodynamic parameters, including velocity, wall shear stress (WSS), pressure, vorticity and turbulent kinetic energy (TKE). We find that WSS on the aortic root (AoR) is around two times lower when the OG is attached to the coronal side of the aorta using an angle of 45° ± 10° at a distance of 55 mm.

**Discussion:** The results show that the OG placement may significantly influence the haemodynamic patterns, demonstrating the potential application of CFD for optimising OG positioning to minimise the risk of cardiovascular complications after LVAD implantation.

## 1 Introduction

In the UK, more than 900,000 people are living with heart failure (HF) (NHS, 2022). HF is defined as a structural and/or functional abnormality that produces raised intra-cardiac pressures or inadequate cardiac output at rest or during exercise. Heart transplants are the gold standard option for patients with end-stage HF, but donor organs are a limited resource and only around 200 transplants are performed annually in the UK (BHF, 2023). This highlights the pressing need for alternative treatment options (Shaw et al., 2019).

**FIGURE 1:**
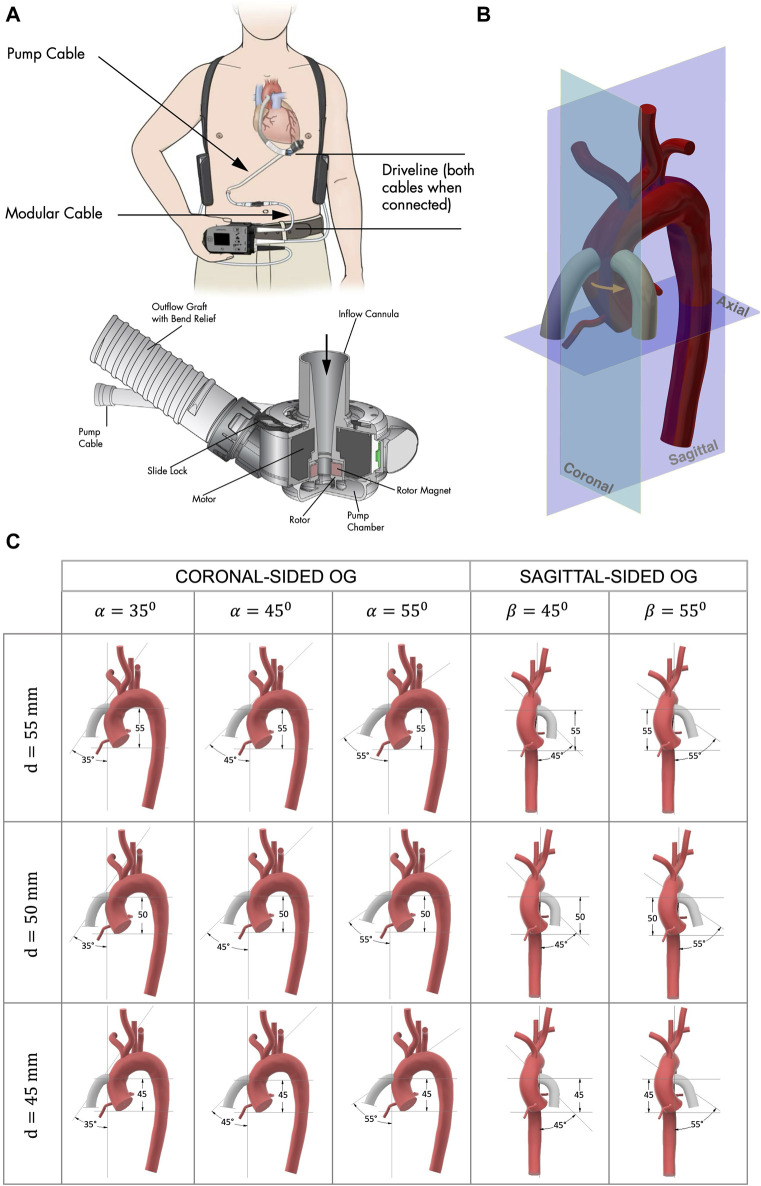
**(A)** HMIII LVAD courtesy of Abbott Laboratories (Abbott, 2017). The titanium pump provides continuous flow and creates a bio-surface with the blood; the inflow cannula drains the blood from the LV apex to the LVAD; the outflow PTFE graft directs the flow from the pump to the ascending aorta; and the extracorporeal controller is connected via a percutaneous driveline. **(B)** The anastomosis of the OG to the aorta, showing the two cardinal positions of the OG around the aorta, coronal sided and sagittal sided, where the patient coordinate system is the same as the coordinate system of the medical imaging. **(C)** Geometrical parameters for the model anastomosis of the HMIII OG to the aorta assessed in this study where *d* is the distance from the AVJ to the OG, α is the angle between the aorta and the coronal-sided OG, and β is the angle between the aorta and the sagittal-sided OG.

Mechanical circulatory support devices are used to help pump blood around the body and were first developed in the 1960s for patients who were unable to be weaned from cardiopulmonary bypass after cardiac surgery (Uriel et al., 2017). Initial mechanical support devices were external systems but there has been major progress in device technology and now fully implantable left ventricular assist devices (LVADs) are under development. LVAD therapies may include short and long-term support for patients with HF. The HeartMate III (HMIII) is the latest clinically approved model, certified in Europe in 2015 (Figure 1A). It was designed to extend the potential for long-term LVAD support by reducing thrombotic and haemorrhagic complications and has resulted in improvements in patient survival and wellbeing (Akan et al., 2014; Bourque et al., 2016).

Research on LVADs has been a multidisciplinary effort using *in vitro* experiments, clinical trials, and numerical simulations, which has helped to improve pump design and patient outcomes (Fraser et al., 2011; Bourque et al., 2016). While engineering methods have always informed LVAD design, there is significant potential to apply these approaches to enhance other procedural considerations including surgical optimisation (Bourque et al., 2022). Computational fluid dynamics (CFD) simulations can be used to analyse how various pump and patient factors affect blood flow in the aorta and investigate the causes of complications that emerge during LVAD support (Grinstein et al., 2021).

The aim of this study is to examine the aortic flow patterns produced by different geometrical parameters of the anastomosis of the HMIII outflow graft (OG) to the aorta. We consider whether the risk of cardiovascular complications could be minimised by optimising the positioning of the OG during LVAD implantation. Medical practitioners have observed that postoperative outcomes for LVAD patients depend greatly on the intraoperative process, including the attachment of device components (Beyersdorf et al., 2017; Peek et al., 2023). Computational studies have quantified how LVAD flow can predispose patients to conditions such as aortic regurgitation (AR) (Vriz et al., 2022; Lopez-Santana et al., 2023; Sahni et al., 2023), but preoperative planning may be able to reduce complications related to flow perfusion and shear stress (Ruiz-Soler et al., 2017; McElroy et al., 2020a; McElroy et al., 2020b; Swanson et al., 2020; Deyranlou et al., 2021; McElroy et al., 2021; Hosseini and Keshmiri, 2022). Preliminary studies with HMII show promising results that modifying geometrical parameters such as the angle of insertion can influence the risk of AR development (Kasinpila et al., 2020; Yoshida et al., 2020). We extend this by offering a detailed consideration of the impact of OG positioning on aortic flow patterns in relation to HMIII, which warrants investigation as, despite improvements in the technology, complications remain prevalent with this model (Imamura et al., 2020; Rali et al., 2022). This study demonstrates that CFD simulations could provide surgical teams with a preoperative tool to optimise OG attachment during LVAD implantation and thus reduce abnormal flow patterns associated with adverse patient outcomes.

## 2 Materials and methods

The haemodynamic parameters selected for the present study are velocity, pressure, vorticity, turbulent kinetic energy (TKE), and wall shear stress (WSS). These are included as parameters of interest because they appear to have the most relevance for adverse effects in patients with LVAD. High velocity and WSS throughout the cardiac cycle result in higher arterial pressures and turbulent flows around the aortic wall (Vriz et al., 2022), producing a shearing effect which can lead to endothelium dysfunction and increased thromboembolic risk (Sciaccaluga et al., 2022). Reverse velocity, i.e., flow directed towards the aortic root (AoR) promotes increased flow recirculation and turbulent eddies in the AoR region. These effects are associated with AR, which occurs when blood flows backwards through the aortic valve (AV), as well as other complications such as thrombotic stroke and atherosclerotic disease (Kasinpila et al., 2020). Approximately 25% of LVAD patients develop moderate or severe AR within 1 year of implantation, rising to more than 30% by 2 years (Calin et al., 2022). The incidence and severity of AR increases with the duration of support and results in increased hospitalisation and decreased survival, suggesting that AR is a time-dependent, progressive and clinically significant complication (Deo et al., 2014; Tanaka et al., 2020; Cowger, 2023).

The Reynolds-Averaged Navier-Stokes (RANS) method is applied to simulate the aortic flow, using the CFD software Simcenter Star CCM+, Version 2021.2 (16.04.007-R8, Siemens Digital Industries Software, Plano, TX, USA). The aortic flow is assumed to be incompressible, so that the blood density and volume do not change when subjected to high pressure gradients. Blood is modelled as a Newtonian fluid with a dynamic viscosity of 0.004 Pa s and a constant density of 1,060 kg/m^3^.

### 2.1 Geometry

The surgical procedure of LVAD implantation has some fixed parameters, such as the pump design (Figure 1A). However, a significant factor that can be altered by the surgeon is the OG position. To optimise this, a simplified three-dimensional model of a HMIII OG attached to an aorta is designed (Figure 1B).

Image processing from a 2D PC-MRI scan from a healthy volunteer in their thirties (Deyranlou et al., 2020) is used for the three-dimensional anatomical model of the aorta. A healthy aorta offers an appropriate basis for this study because, although many patients will receive an LVAD after prolonged heart conditions, LVAD can also be offered to patients in the case of a sudden viral infection that could cause acute myocarditis (Schultz et al., 2009) and posteriorly dilated cardiomyopathy (Arbustini et al., 2001). It is important to consider that human aortic anatomy and arch configurations are naturally varied, so the optimal positioning of the OG in any case will be specific to the geometry of the patient’s aorta (Fatona, 2017) and the physiological conditions of each aorta can influence the generation of reverse flow (Bensalah et al., 2014). The aortic arch (AoA) configuration used in this study is the most common (Type I) and does not have any prominent geometrical features such as unusual branch connections, which makes it a useful case study for assessing the aortic flow patterns produced by different OG positionings.

Different geometrical parameters are considered to determinate the best possible combination of the following geometrical parameters (Figure 1B): (i) the distance from the anatomic ventriculo-arterial junction (AVJ) to the OG, *d*, measured using two parallel planes of the axial axis through the furthest point of AVJ and the central point of the OG; (ii) the cardinal position of the OG around the aorta, coronal or sagittal side; and (iii) the angle between the aorta and the OG, α for the coronal and β for the sagittal side, where α is the angle between the coronal plane of the aorta and the axis line of the OG and β is the angle between the sagittal plane of the aorta and the axis line of the OG. A three-dimensional volume-rendered cardiac CT of a patient with a HMIII LVAD (Aghayev and Mehra, 2019) is used as a starting point to estimate the initial parameters of attachment. The OG is located at approximately *d* = 50 mm and α = 45° and with this information a prototype of the anastomosis of the OG to the aorta is modelled using Computer-Aided Design (CAD) software.

We identified the limits for each parameter that could be included for this case study based on the aortic geometry and the appropriate surgical technique in a clinical context. The positioning of the HMIII OG in the surgical procedure is restricted due to the diameter of the OG (14 mm), which is larger than some alternative LVAD models such as the HeartWare Ventricular Assist Device (10 mm) (Bourque et al., 2022). The geometrical parameters determined as suitable for this patient are the angles in a range between 45° ± 10° for the coronal-sided OG and between 45° and 55° for the sagittal side and the distances between 50 ± 10 mm for both cardinal positions, resulting in 15 key combinations for comparative assessment (Figure 1C).

### 2.2 Mesh

The polyhedral mesh from the commercial software Star CCM+ is used for the simulation of the haemodynamic flow (Spiegel et al., 2011). The tetrahedral cells are considered less accurate for the finite volume method due to their pyramidic shape in comparison with the polyhedral cells which attempt to approximate a sphere. The meshes used for the simulations in this study (Table 1) include a surface mesh preparation with a re-mesher, followed by a polyhedral mesher, and finally a mesh refinement with the prism layer mesher. In line with standard CFD practice for refining the mesh around the wall surfaces to accurately capture the boundary layer, smaller polyhedral cells are used at the vessel wall, while larger cells are admissible in the central part of the artery, i.e., where the variation of velocity profiles is smaller. Supplementary Figure S3 and Supplementary Table S2 show a mesh study undertaken to validate the cardiovascular flow by comparing the volume flow rate with 4D flow PC-MRI of a healthy aorta which shows a close agreement between the simulation results and the clinical data (Deyranlou et al., 2020).

**TABLE 1:** Comparative table of polyhedral mesh with five prism layers refinement.

	Healthy aorta						Aorta with HMIII LVAD OG
Grid	Coarse (4)		Medium (3)	Fine (2)		Finest (1)	(3)
Base Size (m)	4 × 10^−4^		3 × 10^−4^	2 × 10^−4^		1 × 10^−4^	3 × 10^−4^
Number of Cells	1.198 × 10^6^		2.454 × 10^6^	5.330 × 10^6^		11.011 × 10^6^	3.064 × 10^6^
Number of Faces	5.811 × 10^6^		11.990 × 10^6^	24.561 × 10^6^		54.330 × 10^6^	15.037 × 10^6^
Number of Verts	3.998 × 10^6^		8.297 × 10^6^	16.236 × 10^6^		37.843 × 10^6^	10.438 × 10^6^
Minimum Wall Y+	2.568 × 10^−4^		1.823 × 10^−4^	1.106 × 10^−4^		0.547 × 10^−4^	1.924 × 10^−4^
Maximum Wall Y+	6.992 × 10^−1^		5.081 × 10^−1^	3.941 × 10^−1^		3.134 × 10^−1^	7.789 × 10^−1^
Use of Memory (MB)	278.20		423.96	818.91		1764.89	503.89

**FIGURE 2:**
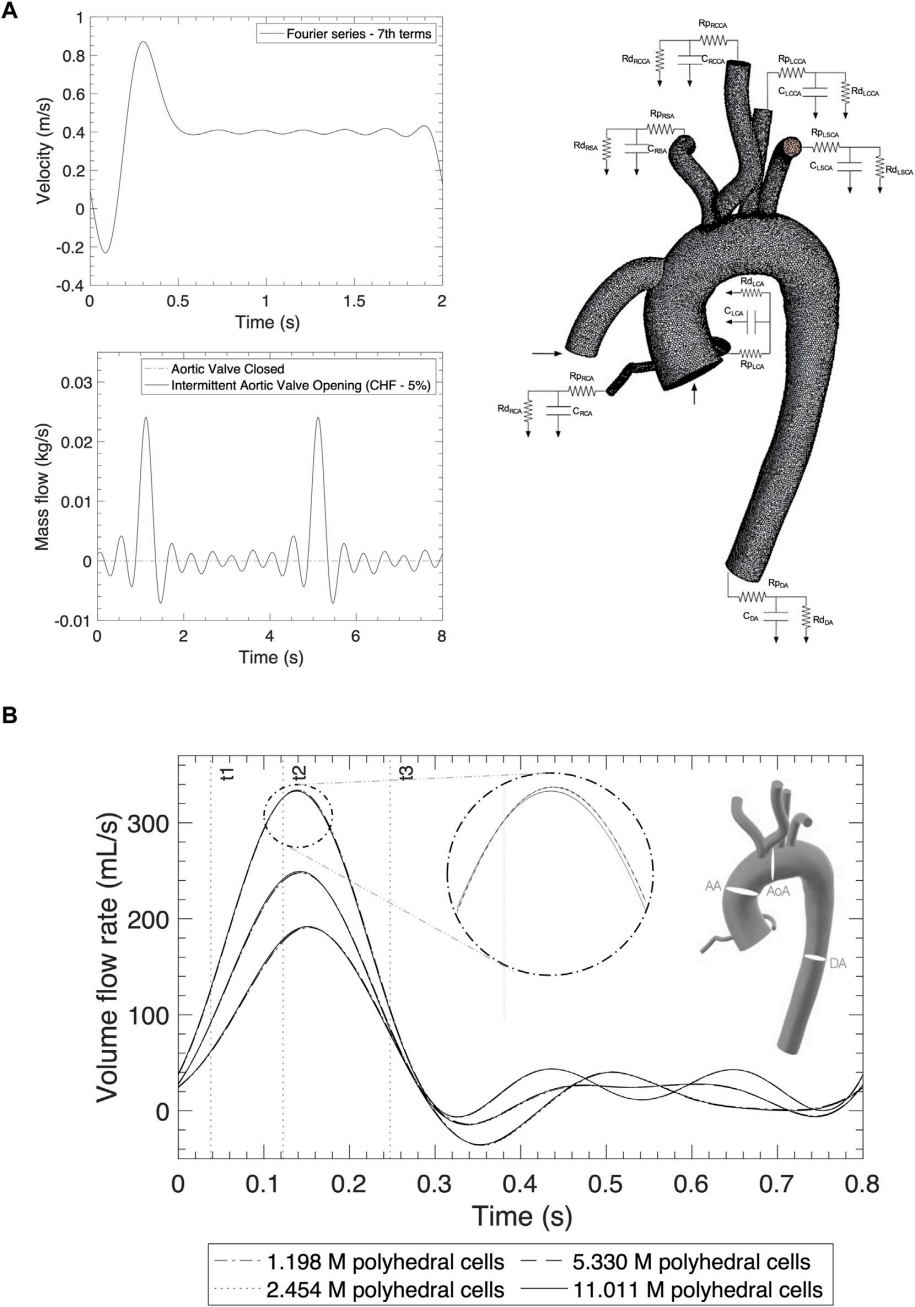
**(A)** Inlet profiles for the AoR and OG and outlet boundary conditions applying the Windkessel model. **(B)** Mesh independency comparison of the volume flow rate from different sections: the ascending aorta, aortic arch and descending aorta for a normal cycle.

Comparison of the volume flow rate and WSS for all the meshes are notably consistent regardless of grid resolution (Supplementary Figures S3, 4). A subtle, yet important, pattern variation is at the peak systolic where the highest computed quantities slightly differ and is studied in more detail in Table 2. Figure 2B shows the three different cross-sections that are placed in strategic positions in the ascending aorta (AA), the AoA, immediately after the right common carotid artery (RCCA), and the descending aorta (DA), as well as the volume flow rate against time for one cycle of 0.8s.

**TABLE 2:** Grid refinement study-summary of Richard extrapolation.

	Velocity - AA section		WSS - aortic wall	
	*p* = −9.033		*p* = 2.337	
	E	GCI (%)	E	GCI (%)
Finest (1)–Fine (2)	−3.047 × 10^−3^	0.381	0.008	1
Fine (2)–Medium (3)	−2.840 × 10^−4^	0.036	0.016	2

Table 2 shows a mesh convergence analysis through a more quantitative approach based on the calculation of the grid convergence index (GCI) for the maximum volume flow rate and WSS (Craven et al., 2009). The GCI percentage is directly proportional to the percentual error and is 10 times higher for the volume flow rate for the medium to fine meshes while the WSS values improve by 1% with the fine to finest meshes. Both GCI are in the asymptotic range of convergency, demonstrating that the solution is mesh independent. One of the parameters to consider for applicability is to avoid a significant increase in memory usage and generation time, which is directly proportional to the cost. On this basis, the parameters of the medium grid with 2.5 million cell polyhedral mesh with five levels of prism layer refinement are adopted to simulate the positioning of the HMIII OG attached to the aorta. The introduction of the OG artificially attached to the aorta increases the number of cells by 25% and the memory usage by 19%.

### 2.3 Boundary conditions

#### 2.3.1 Inlets

The simulation is designed with two inlets, the OG and the AoR, which are described as the artificial pulse from the HMIII pump and the native cardiac cycle respectively. The HMIII LVAD has a unique feature known as artificial pulse, which is reproduced in the transoesophageal echocardiography analysis of the OG inlet profile (Sidebotham et al., 2018). At insertion, the clinician sets up the HMIII LVAD at a fixed speed, and the software algorithm of the pump produces an artificial pulse based on variations in speed in a repetitive cycle every 2 s. The pump decreases its initial fixed speed by 2000 rpm for 0.15 s, followed by an increase of 4,000 rpm for 0.2 s, before returning to the initial speed for 1.65 s. The typical range of rotational speeds for the HMIII LVAD varies between 5,000 and 6,000 rpm, 83.3–100 Hz (Loring et al., 2020).

The inlet velocity profile for the OG which represents the artificial pulse of the pump (Figure 2A) is obtained from the cardiovascular imaging of the continuous-wave spectral doppler echocardiographic detention of the HMIII OG velocity in Magunia et al. (2017) and the Fourier series approximation.

$$V\left(t\right)=a_{0}+\sum_{n=1}^{n=8}a_{n}\times \cos\left(n\times \omega \times t\right)+\sum_{n=1}^{n=8}b_{n}\times \sin\left(n\times \omega \times t\right)$$

where a and b are the coefficients of the series, n is the number of terms in the series, ω is the fundamental frequency of the signal and $a_{0}$ mimics a constant or interception term in the equation and is related with the cosine term when n=0 .

The inlet boundary condition for the entrance of the AoR is simulated using two conditions of AV flow, when it opens intermittently and when it is closed (Figure 2A). This study focuses on the case where the AV opens intermittently because, depending on the state of the LV and the patient’s residual native contractility, the LVAD may function alongside the native cardiac output and result in intermittent opening of the AV (Mahr et al., 2017). For the intermittent cycle, a mass flow time-dependent function is derived using data from a simulation of chronic HF in the Harvi software (Version 2.1.4) (Burkhoff et al., 2021) with a cardiac output of approximately 3.2 L/min and represented by the Fourier series in cycles of 0.7s (Leisman and Burkhoff, 2017).

The native cardiac output towards the AoR after LVAD implantation may vary depending on HF severity and other physiological conditions (McElroy et al., 2016; McElroy et al., 2020a). The asynchronous opening of the AV can induce different flow patterns depending on the state of the LV which is patient specific. The simulations in this study ascribe 5% of remnant cardiac output as the residual flow through the AoR (Callington et al., 2015), with a frequency, which depends on the assumed opening of the AV, of once every five cycles (Aliseda et al., 2017).

#### 2.3.2 Outlets

The aorta is assumed to be rigid with a pressure outlet in each artery modelled using the Windkessel model (Sridharan et al., 2012; Black et al., 2023) (Figure 2A). In Star CCM+, a three-element WK model can be represented by adding Ordinary Differential Equations in the field functions (Siemens Community, 2020). The calculation of the Windkessel conditions is detailed in the Supplementary Material.

The RCR values for each artery (Table 3) are calculated by relating the haemodynamic parameters to a circuit in parallel, where the proximal resistance is related to a viscosity resistance, the distal resistance is related to the resistance of capillaries and veins, and the capacitor is equivalent to the vessel compliance. For the HMIII LVAD, constants calculating the following values were assumed: systolic pressure, 78 mmHg; diastolic pressure, 69 mmHg; mean arterial pressure, 73 mmHg; cardiac output, 6.7 × 10^−5^ m^3^/s; heart rate, 30 BPM; and aortic distensibility, 7.9 × 10^−3^ mm/Hg. For this simulation, the velocity and mass flow rate distribution are used for the inlets and resistances and the compliance parameters of the Windkessel model are applied at the outlets.

**TABLE 3:** Windkessel constants for the anastomosis of the HMIII LVAD OG to the aorta.

	Rp (Pa s m^-3^)	Rd (Pa s m^-3^)	C (m^3^ Pa^−1^)
Right Coronary Artery	5.42 × 10^8^	7.17 × 10^9^	2.12 × 10^−10^
Left Coronary Artery	5.42 × 10^8^	7.17 × 10^9^	2.12 × 10^−10^
Right Subclavian Artery	7.79 × 10^7^	1.03 × 10^9^	1.47 × 10^−9^
Right Common Carotid Artery	7.38 × 10^7^	9.76 × 10^8^	1.55 × 10^−9^
Left Common Carotid Artery	1.21 × 10^8^	1.61 × 10^9^	9.45 × 10^−10^
Left Subclavian Artery	1.06 × 10^8^	1.41 × 10^9^	1.08 × 10^−9^
Descending Aorta	2.01 × 10^7^	2.66 × 10^8^	5.70 × 10^−9^

#### 2.3.3 Wall

For the blood viscous flows, the no-slip condition for viscous fluids assumes that at a solid boundary, the fluid will have zero velocity relative to the boundary.

### 2.4 Numerical framework

The RANS time-averaged equations provide velocity and pressure information for the aorta and branches at any point in time and location. Since the haemodynamic parameters of the flow in the arteries cannot be measured directly, a computational method that solves the equations to describe the motion of the fluids, Navier Stokes equations, is used:

$$\frac{\partial \rho }{\partial t}+\frac{\partial \left(\rho u_{i}\right)}{\partial x_{i}}=0$$

$$\frac{\partial \left(\rho u_{i}\right)}{\partial t}+\frac{\partial \left(\rho u_{i}u_{j}\right)}{\partial x_{j}}=-\frac{\partial P}{\partial x_{i}}+\frac{\partial }{\partial x_{j}}\left(\mu \frac{\partial u_{i}}{\partial x_{j\;}}\right)$$

where u is the velocity, P is the pressure, ρ is the density and μ is the dynamic viscosity. The discretization of the partial derivative equations at the grid points uses the finite volume method with a time-step of 0.001 s.

The pressure-velocity coupling procedure is the Pressure Implicit with Splitting of Operators algorithm, which was proposed for non-iterative computation of unsteady compressible flow but has been adapted for wider applications such as cardiovascular flow circulation (Versteeg and Malalasekera, 2007).

Turbulent flow can occur in the ascending aorta in subjects with normal cardiac function and this can become constant for individuals with AV abnormalities (Stein and Sabbah, 1976; Yoshida et al., 2020). A turbulence model comparison is provided in the Supplementary Material which shows that the Realizable k−ω SST with two-layer all-y^+^ turbulence model gives a better approximation of the aortic flow for this specific case study. There is a debate over the choice of turbulence model for cardiovascular flows that include a heart pump (Fraser et al., 2011). While the flow is mainly laminar, it can transitionally become turbulent, notably at the artificial peak systolic (Re > 5,000) due to the high-velocity fluctuations induced by the centrifugal pump. The k−ω SST turbulence model tends to generate some of the most accurate approximations for simulating cardiovascular flow during LVAD (Ghodrati et al., 2021) and for describing the flow behaviour of centrifugal pumps (Bai et al., 2022). The use of two-layer wall treatment adds the flexibility of an all-y^+^ wall treatment. In the comparison of the computational accuracy of the three turbulence models, it can be observed that the k−ϵ turbulence model overestimates the values because the aortic flow is not in the fully turbulent regime (Mao et al., 2020). The vortices in the turbulent flow dissipate energy due to their recirculating motion (Salman and Yalcin, 2021) and fluid layers mix via eddies and swirls. The energy loss can be estimated from the turbulent velocity gradients (Wang et al., 2021), which provides an additional indication of whether some OG placements are more efficient than others.

$$Loss=\int_{t_{1}}^{t_{2}}\frac{\mu }{2}\sum_{\forall_{i,j}}{\left(\frac{\partial u_{i}}{\partial x_{j}}+\frac{\partial u_{j}}{\partial x_{i}}\right)}^{2}dt$$

In periodic unsteady simulations, one of the criteria to assess the convergence of the solution is to check whether the monitor plots of the parameters of interest, i.e., the pressure on the outlets, reach stability. This point can be identified by observing the time histories of various flow quantities and ensuring that the transient behaviours observed initially in the simulation are no longer present, verifying whether there is a repeating pattern. The simulation stabilises and follows the same tendency approximately in the fifth cycle with the parameters established previously. Each cycle has a duration of 2 s due to the HMIII LVAD artificial pulse.

## 3 Results

**FIGURE 3:**
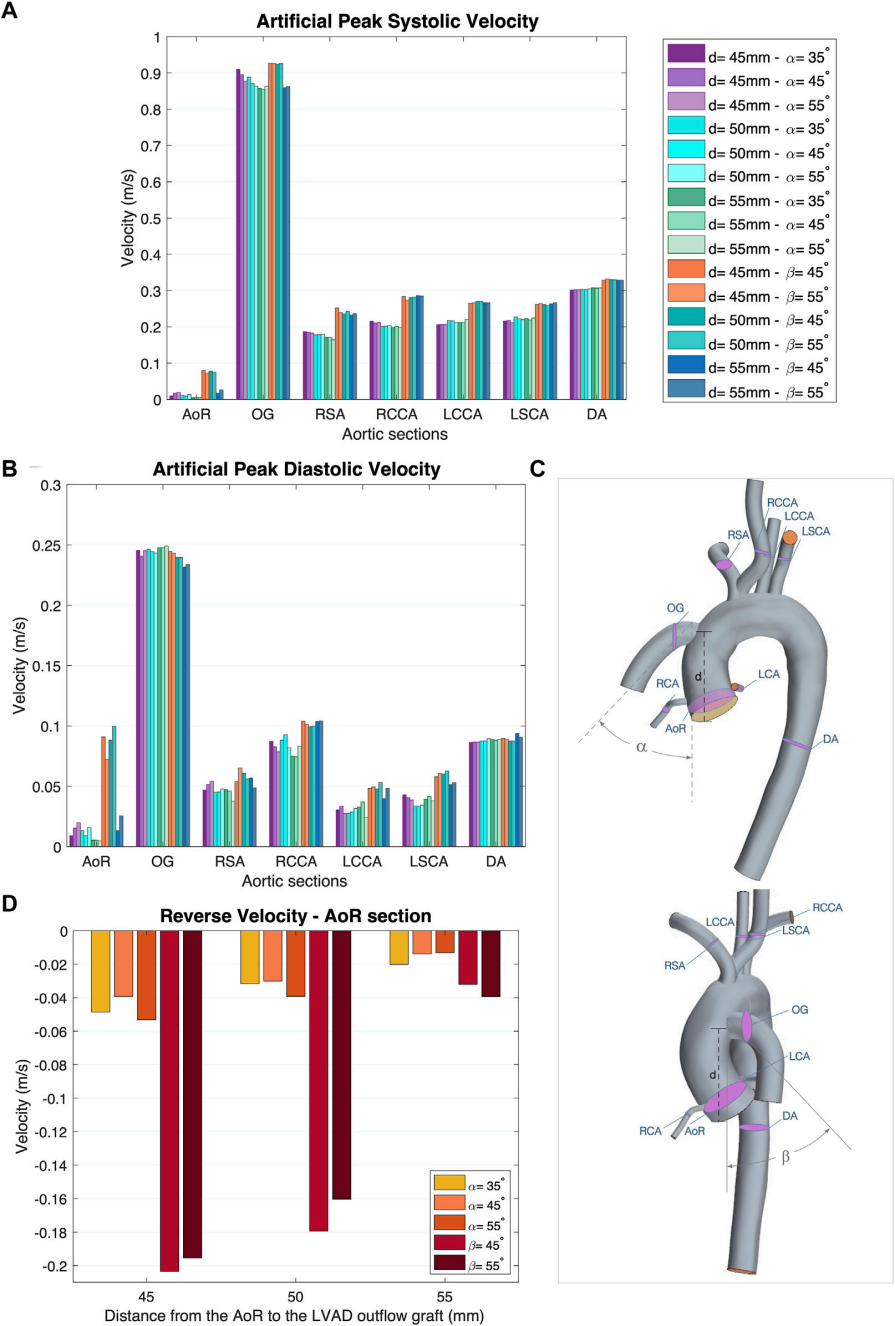
**(A)** Artificial peak systolic velocity. **(B)** Artificial peak diastolic velocity. **(C)** Aortic sections of the coronal- and sagittal-sided OG. **(D)** Reverse velocity (directed towards the aortic root) for the different geometrical combinations for the positionality of the OG.

**FIGURE 4:**
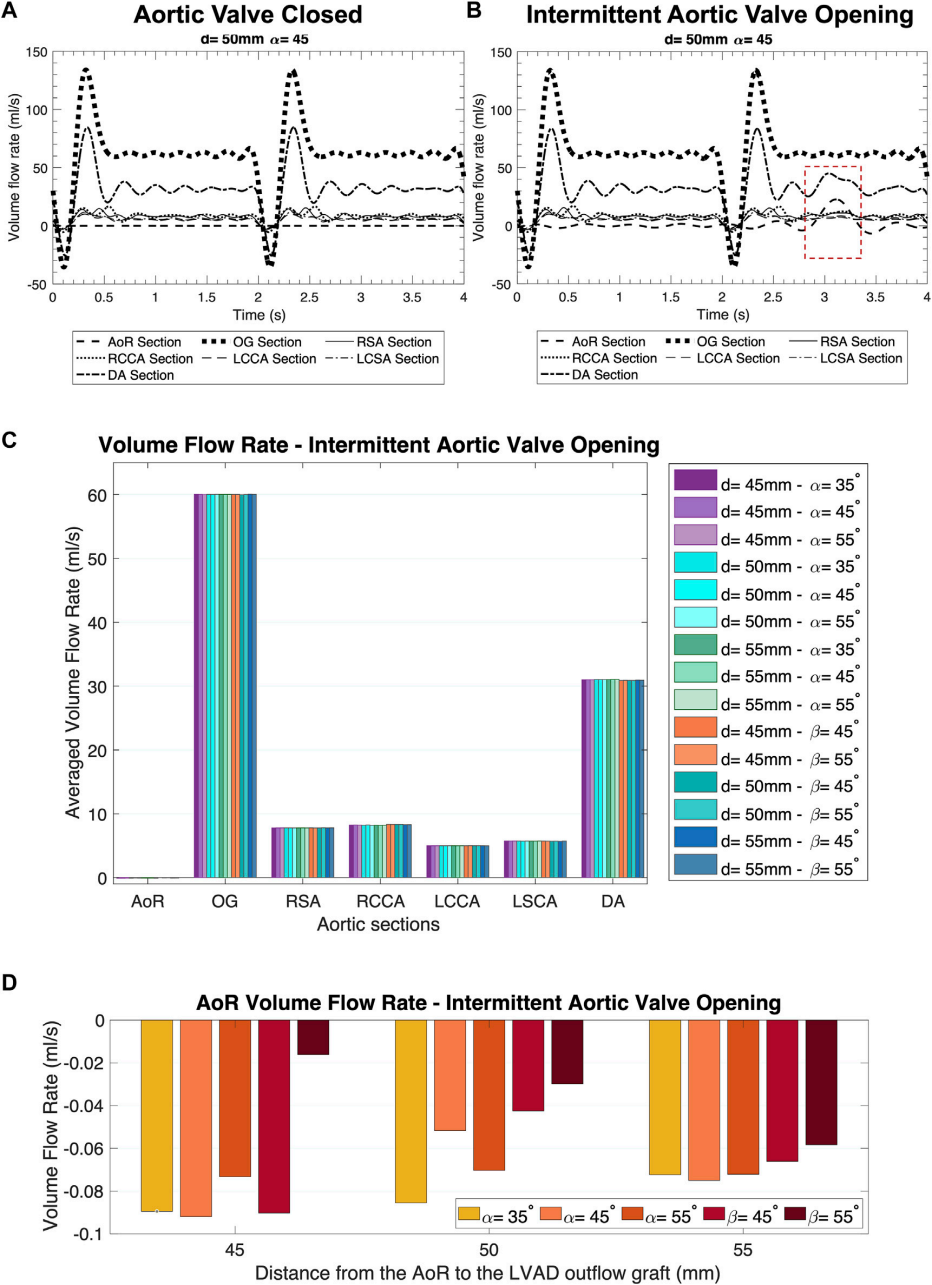
Distribution of the volume flow at the different sections of the aorta over two cycles of the heart pump. **(A)** AV Closed inlet condition. **(B)** Intermittent AV Opening inlet condition. **(C)** Averaged volume flow rate comparison in the coronal- and sagittal-sided OG for the intermittent AV opening. **(D)** AoR flow rate comparison in the coronal- and sagittal-sided OG for the intermittent aortic valve opening.

**FIGURE 5:**
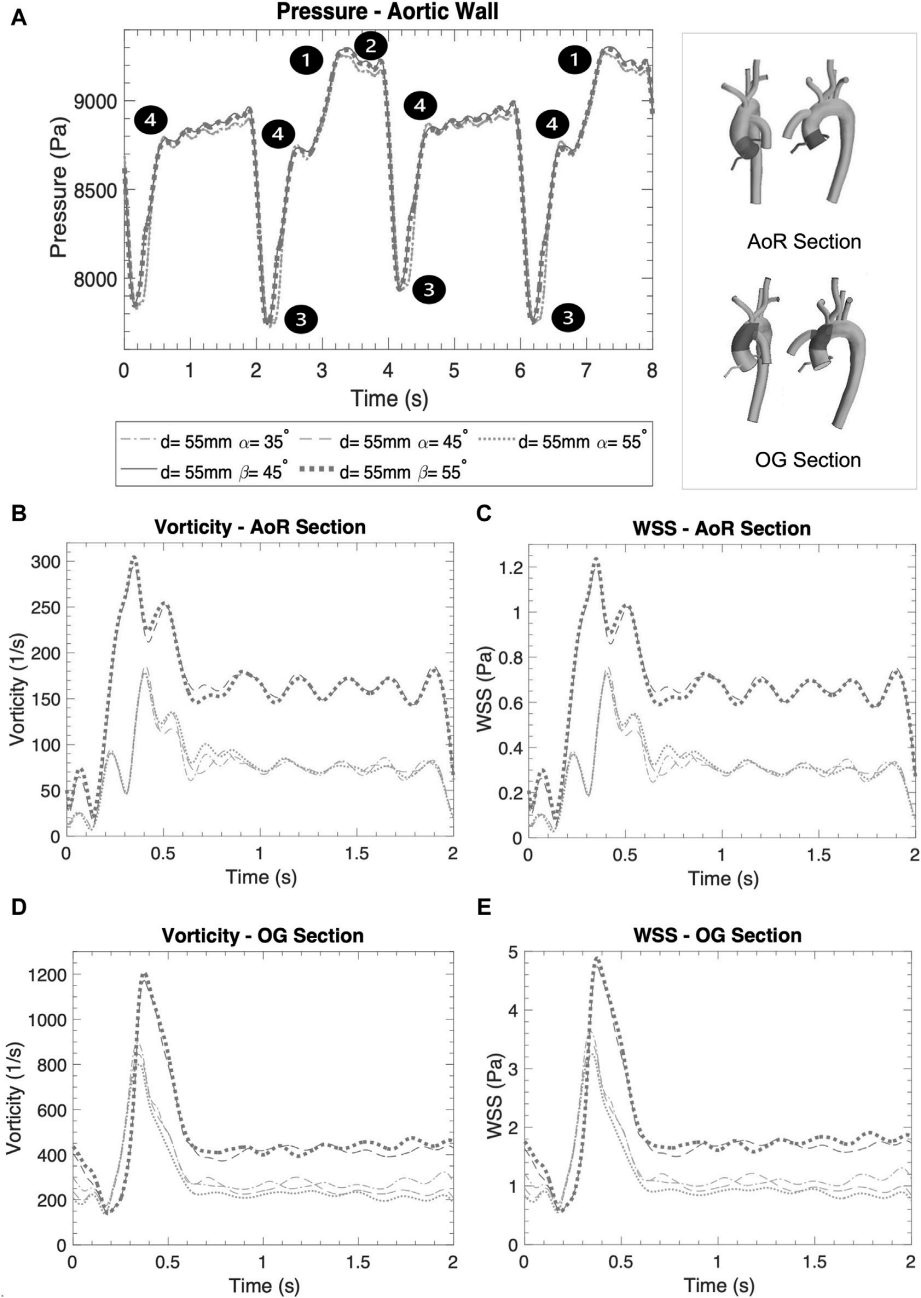
**(A)** Averaged pressure along the entire aortic wall. **(B)** Vorticity in the AoR section. **(C)** WSS in the AoR section. **(D)** Vorticity in the OG section. **(E)** WSS in the OG section.

The postprocessor used to validate the results is the Star CCM + integrated package for CFD data which presents reports of the model at instant times and time-history monitors to review the scalar and vectorial scenes at specific locations through the cycle. The post-processing for this study comprises the creation of reports using quantitative and qualitative data, including section and vector plots, velocity streamlines, area-averaged values, and correlation between parameters (Figures 3–5).

**FIGURE 6:**
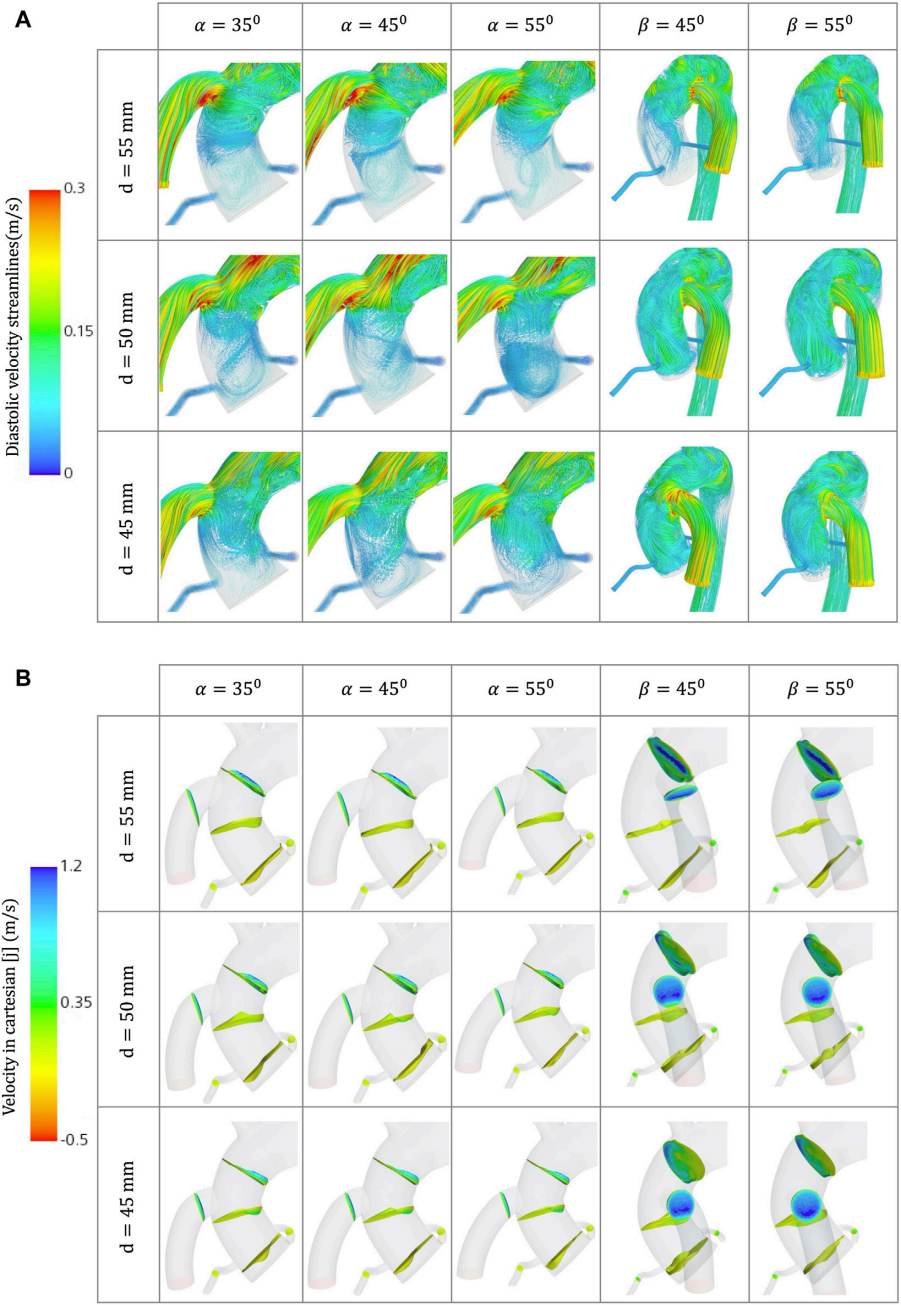
**(A)** Velocity streamlines for the different geometrical combinations for the positioning of the OG at artificial peak diastolic. **(B)** Velocity profiles comparison for the different geometrical combinations for the positioning of the OG at artificial peak systolic.

**FIGURE 7:**
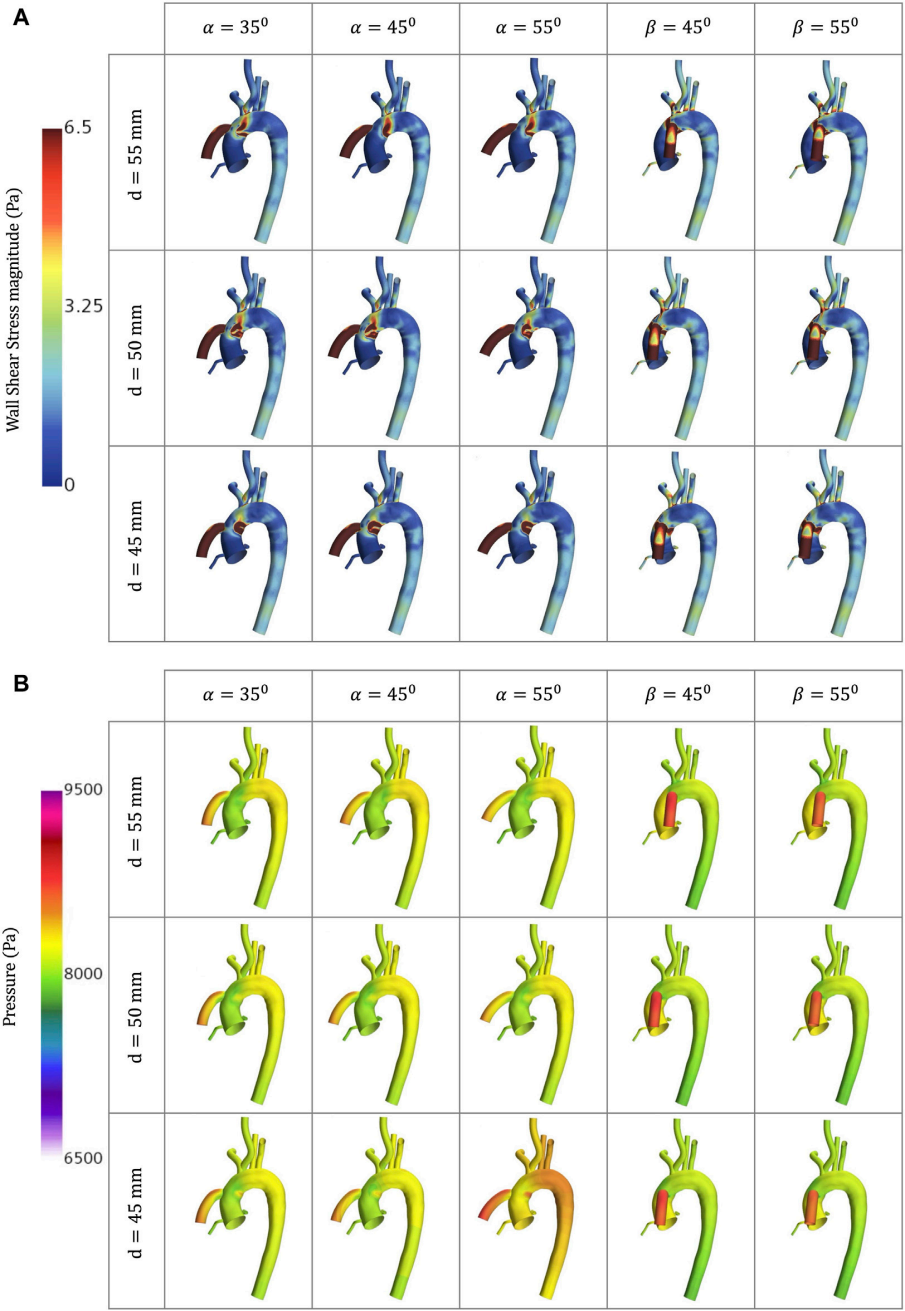
CFD simulations for the different geometrical combinations for the positioning of the OG at artificial peak systolic representing, representing: **(A)** WSS; and **(B)** Pressure.

**FIGURE 8:**
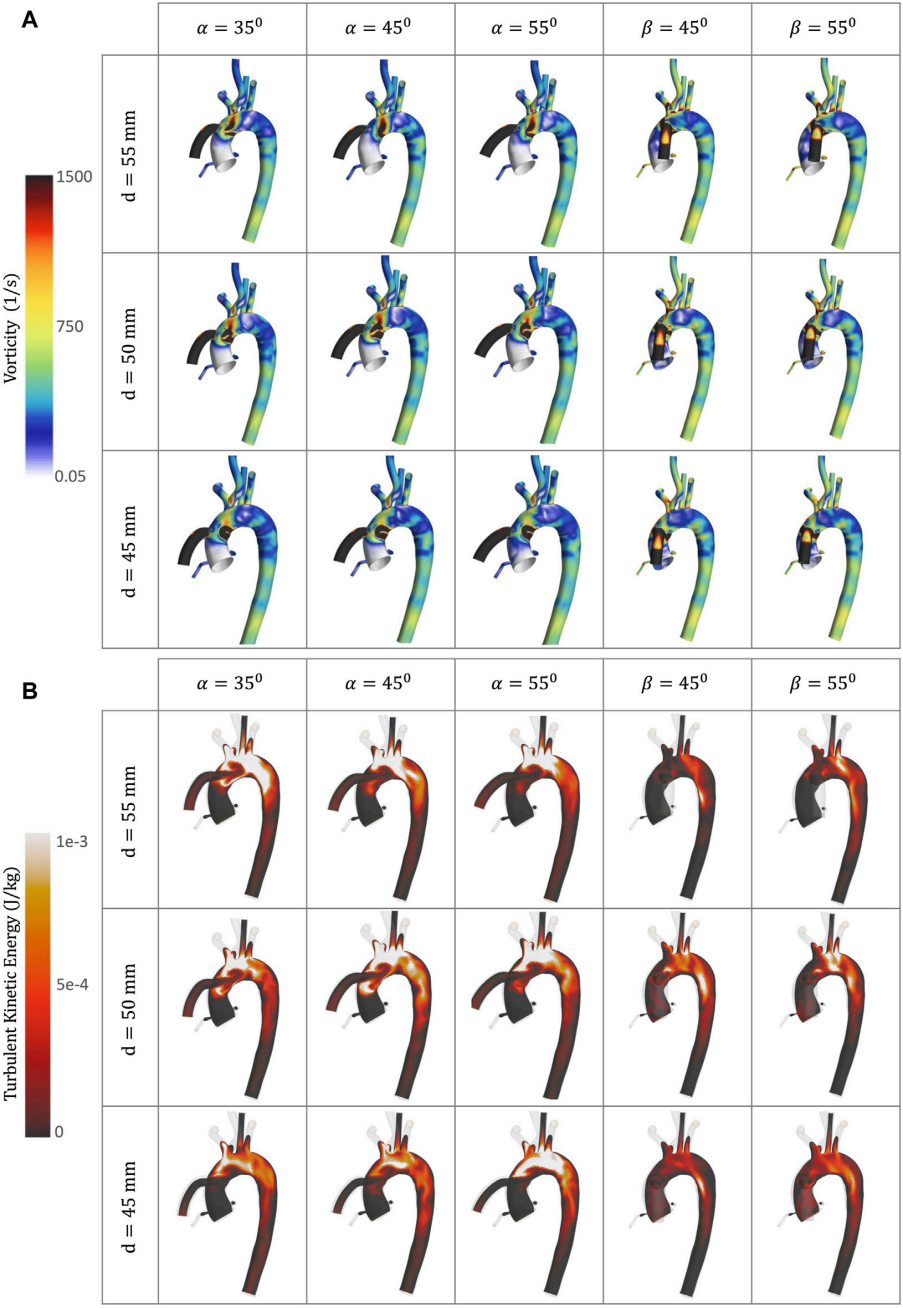
CFD simulations for the different geometrical combinations for the positioning of the OG at artificial peak systolic, representing: **(A)** Vorticity; and **(B)** TKE.

**FIGURE 9:**
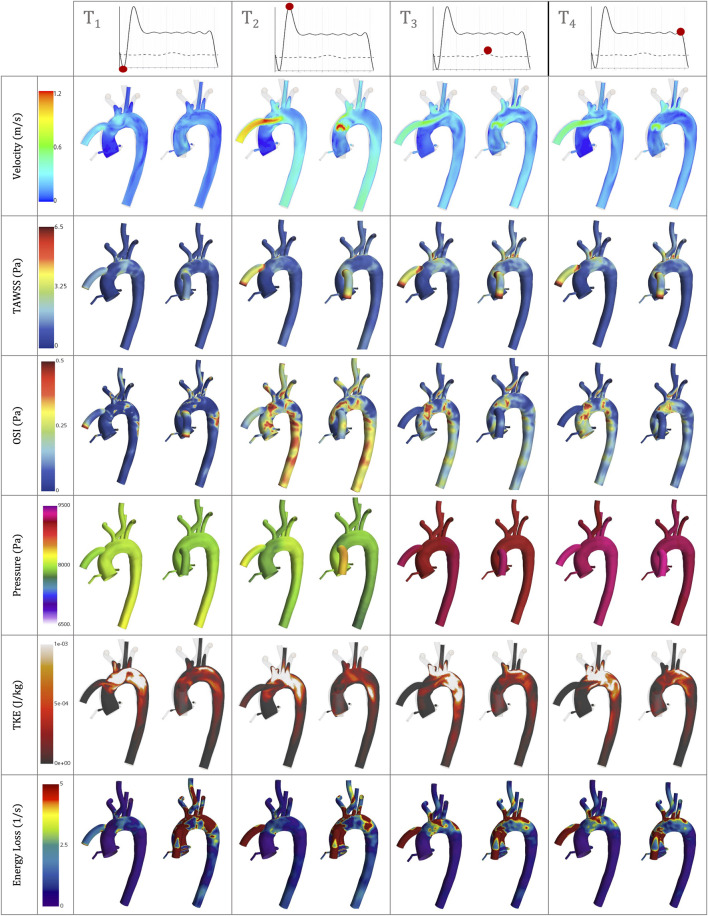
Velocity, TAWSS, OSI, Pressure, TKE and Energy loss simulation results for the best- and worst-case scenarios, the coronal-sided OG (*d* = 55mm, α = 55) and the sagittal-sided OG (*d* = 45mm, β = 45) respectively, at the following time references: peak HMIII artificial diastolic, peak HMIII artificial systolic, peak native cardiac systolic and the end of one cycle of the HMIII.

**FIGURE 10:**
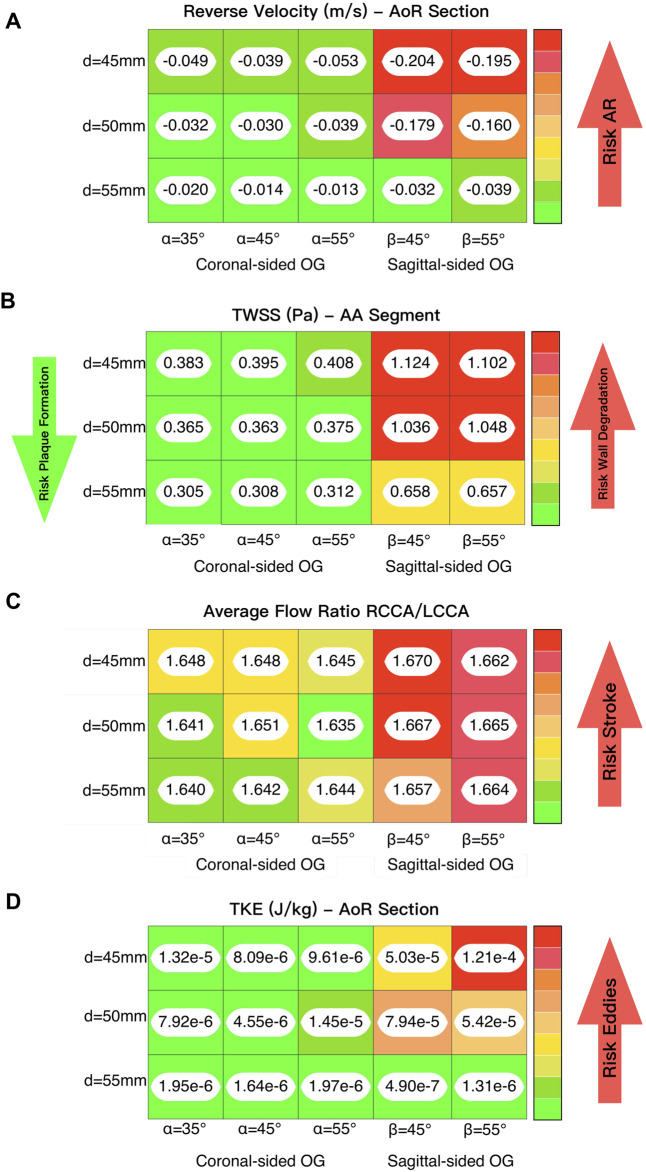
**(A)** Lighting analysis to evaluate the reverse velocity and its correlation with the risk of developing AR. **(B)** Lighting analysis to evaluate the time averaged WSS and its influence on lumen degradation and/or thrombus formation. **(C)** Lighting analysis to evaluate the average flow ratio between the two carotid arteries and its influence on the risk of stroke. **(D)** Lighting analysis to evaluate the TKE and its association with the formation of eddies.

Figures 6–8 offer visual representations of the instant velocity streamlines at artificial peak diastolic, three-dimensional velocity profiles, WSS, pressure, vorticity and TKE in a cardiac cycle at artificial peak systolic. Comparisons between the geometrical parameters demonstrate that the coronal-sided position is preferable for flow perfusion in this specific case. It shows that, in the sagittal position, there is higher velocity and WSS in the carotid and subclavian arteries due to the physical proximity of the OG to these arteries in this position. Figure 9 depicts a deeper study of the fluid behaviour for the best- and worst-case scenarios across the 15 case studies and compares them over the course of the pump cycle at different time references, focusing on velocity, Time-Averaged Wall Shear Stress (TWSS), Oscillatory Shear Index (OSI), pressure, TKE and energy loss. While the best and worst case varies depending on the haemodynamic parameter, the selection is based on a lightning analysis which shows the overall trends (Figure 10). Figure 10 offers a practical guide to the scientific data, drawing out the medical implications of the simulation results in a clear and accessible manner, which is an important step for any interdisciplinary project. It illustrates how different geometrical parameters for the positioning of the OG can affect the aortic flow patterns and demonstrates the utility of CFD as an informative guide for cardiothoracic surgeons seeking to optimise LVAD implantation.

### 3.1 Velocity

Blood flow velocity is one of the most important haemodynamic parameters for evaluating the risk of patients with LVAD developing postoperative conditions. The aorta is divided into nine sections to study the effects of the HMIII OG on aortic flow (Figure 3C). Figures 3A, B show the averaged velocity at the peak systolic and diastolic of the HMIII for the coronal- and sagittal-sided OG and the impact of position on the distribution of flow across the aorta. The blood flow can be considered normal because the artificial peak systolic velocity does not exceed 2 m/s (Bouchez et al., 2019).

The flow patterns vary based on the location of the anastomosis of the OG. The results show that the distance from the AVJ to the OG, *d*, may play a significant role in abnormal physiological AoR conditions after LVAD implantation. When the OG is positioned further away from the AVJ, the velocity jet flow directed towards the AoR section decreases. In this case study, the optimal value of the distance for both the coronal- and sagittal-sided OG is 55 mm. It is important to note that the positioning of the OG changes the velocity distribution between sections. When the OG is placed closer to the AVJ, it increases the flow velocity in the AoR section but the values of velocity to the carotid and subclavian arteries remain uniform at peak artificial systolic (Figure 3B), which suggests the presence of flow recirculation. The highest reverse velocity is produced immediately after the artificial peak systolic from the HMIII OG across all the geometrical combinations considered. Figure 3D represents the reverse velocity in the direction of the AoR in bars, distinguishing the higher reverse velocity closer to the AV at *d =* 45 mm for both cardinal positions. The findings show some variation in the optimal angles for α and β, but this has a much less significant impact on the velocity than the distance.

Additionally, at peak artificial diastolic, the velocity streamlines vary in direction and magnitude (Figure 6A), indicating the tendency to form eddies, which can be caused by unsteady flows produced, for example, by the close curvature of the aorta and the changes of angular velocity of the heart pump (Torner et al., 2021). The aortic velocity below the coronal-sided OG is higher in the direction of the AoR but produces two sharply curved patterns that collide with the lumen and then turn around upwards in the direction of the AoA and downwards in the direction of the AV (Figure 6A). The same occurs in the AoR section where some of the flow is reversed towards the coronal-sided OG. The sagittal-sided OG has a more uniform linear pattern of flow towards the AV which appears to produce fewer eddies around the AoR (Figure 10D), however the velocity is higher. The TKE is higher in the proximal sections to the OG and the arteries due to the eddies formed by the OG jet flow inlet, especially around the vessel bifurcations (Figure 8B). The three-dimensional velocity profiles at the AoR section for both cardinal positions (Figure 6B) show an almost flat velocity profile when the flow enters the aorta but a subsequent presence of reverse velocity. This reverse flow is distributed around the section in an irregular shape for the coronal-sided OG while, in the sagittal side position, it is concentrated within the centre of the AoR when the distance is 45mm, which may raise the risk of AR development.

### 3.2 Volume flow rate

The volume flow rate is important for depicting how the flow patterns operate in the LVAD circuit and therefore for assessing how to optimise its positioning (Grinstein et al., 2021). Figures 4A, B present the surface averaged volume flow rate for different regions of the aorta and OG when the inlet conditions mimic the AV closed and intermittent opening where the native cardiac cycle is combined with the HMIII LVAD inlet during the unloading of the LV, with a prominent diastolic velocity produced by the fixed speed in the HMIII pump. A positive disturbance in the wave produced by the AV opening (Figure 4B) indicates that the aortic flow is directly influenced by the remnant flow of the native cardiac cycle for the coronal-sided OG at *d =* 45 mm and α = 45°, illustrating the tendencies observed across all the geometrical combinations.

The mass flow rate directed towards the AoR section, just after the OG inlet, should be significantly less than the mass flow rate towards the rest of the arteries, as this may reduce the risk of AR development (Figures 4C, D). A close circulatory loop, where the flow from the LVAD OG is recirculated rather than distributed to the rest of the body, can occur in the presence of significant AR. Although the flow coming from the AV is negligible, it can be observed that even a small opening of the AV makes a high impact in the mass flow rate (Figure 4B), which has a direct influence on other haemodynamic parameters. The volume flow rate bars in the AoR section at intermittent AV opening signify flow directed towards the AV, thus depicting differences in reverse flow for different geometrical positions (Figure 4D).

### 3.3 Wall shear stress, time-averaged wall shear stress and oscillatory shear index

The WSS is the tangential force exerted by flowing blood along the artery lumen. Higher values of WSS (>7 Pa) is a significant factor in the deterioration of the vascular wall over time, causing endothelial lesions and platelet activation (Dolan et al., 2013; Kabinejadian et al., 2016; Gijsen et al., 2019; Xenakis et al., 2023). Depending on the severity of the variation of pump operation, this could also produce plasma skimming, especially at the bifurcations (Nissen and Sirs, 1972; Wiegmann et al., 2019). Low WSS (≤1 Pa) is associated with the development of atherosclerotic plaque and it can be difficult to predict whether the subsequent effect will be plaque rupture or progression as this depends on the patient’s local inflammatory response (Gijsen et al., 2019). This activation mechanism has the potential to stimulate thrombus formation (Cameron et al., 2020). TWSS is the time-based average of WSS vector for one cardiac cycle. High values of TWSS indicate the regions where the flow strikes the lumen and separates, while low TWSS means the recirculation of the aortic flow (He and Ku, 1996). OSI evaluates the variations of the WSS vectorial components, showing flow disturbance, and high values of OSI (≈0.5) can confirm the existence of blood recirculation which can lead to aortic complications (Fytanidis et al., 2014; Gijsen et al., 2019).

Figures 5C, E show the WSS magnitude captured in the AoR and OG sections. They indicate that there is no significant variation between different angles for the coronal- and sagittal-sided OG but, for the sagittal side, the WSS for *d* = 45 mm is approximately 1.5 times higher than *d* = 50 mm. Overall, the sagittal-sided OG multiplies the TWSS more than two-fold in comparison with the coronal-sided OG (Figure 10B). The WSS for the sagittal-sided OG remains consistently high and concentrated in the sections around the OG due to its focal position whereas these values for the coronal-sided OG are distributed more evenly around the OG and along the curvature of the AoA during the artificial peak systolic (Figure 7A).

Figure 9 shows the TWSS and OSI for the coronal-sided OG (*d* = 55mm, α = 55) and sagittal-sided OG (*d* = 45mm, β = 45) at different times of the cycle. It can be observed that there is an overall effect on the arterial bifurcations and at the surgical junction with higher values on all the near-wall haemodynamic parameters considered along the cycle. After the flow travels from the OG to the aorta, it is then distributed to each of the branches, mainly depending on their area. These regions present a variable geometry and are largely exposed to disturbed values of WSS. The highest values of TWSS (>6.5 Pa) are presented around the OG anastomosis for both cardinal positions and continue from the proximal AoA until the proximal descending aorta landmarks due to the continuous-flow nature of the heart pump. The higher values of OSI (>0.5) are at the instantaneous artificial peak systolic where the regions of flow recirculation can be observed close to the OG and around the AoR and certain regions of the descending aorta. Once this behaviour stabilises, the OSI remains persistent only next to the OG section. When low WSS (<1) then occurs in the AoR, this region may not have sufficient flow to produce a good washout, especially when the AV remains closed, which can increase the propensity for plaque formation. The LVAD flow induces recirculation, confirmed by the higher values of OSI, which can affect this plaque environment and prompt the risk of AoR thrombosis (Fried et al., 2018). The combination of high and low WSS across the cardiac cycle raises the risk of cardiovascular complications. This delicate environment ideally requires equilibrium, which highlights the need to optimise the positioning of the OG to promote good flow patterns and reduce adverse cardiac events.

### 3.4 Vorticity

Vorticity is generally used to visualise vortical or swirling structures within the flow. In this study, vorticity is analysed in two sections: the vicinity of the OG and the AoR. Figures 5B, D show the vorticity magnitude in these sections over time. The highest value of vorticity is at the artificial peak systolic when the blood pumped by the HMIII OG enters the aorta, but the exact time at which it hits this peak varies according to the cardinal position. The results show that the vorticity in the sagittal-sided OG is more than 70% higher than the coronal-sided OG for the AoR section and 30% higher for the OG section. Figures 5B–E show that the WSS magnitude exhibits the same tendency as the vorticity magnitude, suggesting a directly proportional relationship between the WSS and the vorticity magnitudes along the aortic wall. This quasi-linear relationship reflects the Newtonian behaviour of the fluid when constant blood viscosity is applied in the CFD model.

Figure 8A shows vortical structures resulting from secondary flow at the peak artificial systolic in the vicinity of the OG and the AoA. Higher values of vorticity are observed at the OG anastomosis in the sagittal position and when the distance of the OG is closer to the AoR, indicating intense local rotation. This may increase the risk of disturbance in the direction of the flow, which has the potential to transport flaked aortic plaque and lead to thromboembolic events (Liaquat et al., 2021). The vortical structures can also direct the flow patterns towards the AV (Figure 8A), which has the potential to modify the morphology of the AoR and thus increase the risk of AR development.

High levels of fluctuating vorticity are one of the features of turbulent flow (Ting, 2016). The turbulent energy dissipates into heat due to frictional forces along the arterial lumen which increases the values of energy losses. Figure 9 shows the energy loss due to viscous dissipation and highlights the regions of major drag. The higher values are depicted when the OG is placed on the sagittal position. Rapid changes in area, as occurs with the AoA and arterial branching, and regions of high-velocity gradients, such as the OG inlet, are frequently related with high viscous dissipation and TKE (Figures 8B, 10A) (Iwata et al., 2020). It is important to acknowledge that this is an illustrative study of the results which uses the k−ω SST turbulence model. There is no single model which can entirely capture the numerous three-dimensional eddy-type motions or their random three-dimensional vorticity (Cameron et al., 2020).

### 3.5 Pressure

Blood pressure control is a critical factor for optimising cardiac support for in patients with LVAD (Markham and Drazner, 2013). Figure 5A displays the averaged pressure at the RCCA and demonstrates that the blood pressure profile is unpredictable because the HMIII artificial pulse is a fixed pulse that is out of sync with the native cardiac cycle (Castagna et al., 2017; Ye et al., 2020). Nevertheless, there are four different patterns evident in the data: (1) the systolic native cardiac cycle, (2) the diastolic native cardiac cycle, (3) the HMIII artificial diastolic and (4) the HMIII artificial systolic. The values of the pressure in HMIII are patient-specific and dependent on the LVAD settings, the afterload and pre-existing conditions such as high blood pressure. The main goal is to maintain a mean arterial blood pressure of no higher than 11,999 Pa (90 mmHg) (Markham and Drazner, 2013). For the purposes of this study, the cardinal positions are compared on the higher distance and no significant difference is found between the coronal and sagittal positions for this factor.

The wall pressure represents the normal stresses on the aorta. Figure 7B presents the wall pressure distribution across the aorta, showing that the maximum pressure is concentrated within the OG, especially for the sagittal position, and the pressure reduces as the OG is positioned further away in the aorta. Additionally, Figure 9 shows how the pressure builds progressively over the course of the cycle, with the maximum pressure occurring with the intermittent opening of the AV.

## 4 Discussion

In this study, the geometrical parameters for the positioning of the HMIII OG to the aorta, including distance from the AVJ, cardinal position around the aorta, and angle between the aorta and the OG, have been assessed using the RANS method. The findings of this study show that, in this specific case, the optimal positioning of the OG in the aorta for reducing abnormal reverse flow patterns is *d* = 55 mm using angles between 45° ± 10° for the coronal position.

The risk of complications such as AR development and thrombus formation may be considered lower when the OG is located further from the AVJ (Figure 10A). The angles within this range make a less significant impact on the haemodynamic patterns associated with AR development than the distances, but there is some variation in the optimal angle for each distance and cardinal position. This does not affect all the haemodynamic parameters uniformly, but on average the ideal angle in this case can be considered as 45°. While some of the variations may appear relatively small, they may still be significant due to their cumulative effect over long-term LVAD support.

The attachment of the OG in the sagittal position increases the reverse average velocity in the AoR section and results in an increase in the average WSS and TKE. It also produces higher velocity towards the carotid and subclavian arteries and a jet flow directed towards the centre of the AVJ (Figure 9). This suggests that the coronal-sided OG position may be a better option for reducing the risk of AR (Figure 10A) as well as other potential adverse effects such as thromboembolic stroke (Figure 10C). As outlined above, WSS, at either extreme of too high or too low, can be potentially damaging. Excessively low values can lead to thrombus formation as the lack of flow across the AV may cause stasis. On the contrary, excessively high values can result in wall deformation or the rupture of existing plaque. The optimal positioning has been stated for this case (Figure 10B), but the range for the geometrical parameters should be defined per patient and the values which may be considered too high or low are variable and therefore, need to be analysed in relation to each specific case (Dhawan et al., 2010; Keshmiri and Andrews, 2015; Salman et al., 2019; Zhou et al., 2023).

A number of limitations need to be considered in relation to the results of this study. While 4D flow PC-MRI may offer a more accurate representation of blood flow patterns, it is not possible to obtain this for patients with LVAD because HF patients cannot undergo this intensive experience due to the fragility of their heart. This means that the data available to validate the CFD is limited. In this study, the numerical simulations were validated using an MRI scan of a healthy aorta, but future research could use CT data from patients with LVAD. Given that arterial stiffness has a direct effect on blood pressure (Belz, 1995), accounting for the presence of deformable walls using fluid-solid interaction may improve the depiction of the flow patterns, although previous studies have suggested that fluid-solid interaction analysis, including the motion of the wall of the heart, only accounts for approximately 5% difference in the most widely used metrics such as WSS (Gijsen et al., 2019). Various factors, including the aortic geometry, HF severity, native cardiac output, LVAD speed modulation, and pre-existing conditions such as hypertension and wall dilatation, also warrant further investigation as they may affect the blood flow. An additional factor is the opening of the AV and the condition of the AV leaflets, which influences the flow patterns in the AoR as well as the risk of complications such as AR. Future research could include the AV geometry, although it is important to highlight that, even with advanced imaging techniques, it is difficult to develop a physiologically accurate model of the complex anatomy of the AV and its components (Yoshida et al., 2020) and the AV can remain persistently closed in LVAD patients so AV opening may only have relevance in certain cases (John et al., 2010; Acharya et al., 2023).

LVAD provides a lifeline for patients with end-stage HF, but cardiovascular conditions can develop as complications. Despite the limitations outlined above, the findings of this study suggest that it may be possible to minimise the flow patterns associated with such complications by optimising the OG positioning during LVAD implantation. Each LVAD patient has their own unique anatomy and the potential risk of developing conditions such as AR may be patient specific. However, given that AR is a time-dependent and progressive condition, even relatively small improvements to the associated aortic flow patterns could have a significant bearing on the wellbeing of a patient with LVAD over long-term support. Although we acknowledge that further clinical research is needed to corroborate these findings, we have demonstrated the potential application of CFD for enhancing surgical intervention and reducing the risk of adverse effects associated with LVAD.

## Data Availability

The original contributions presented in the study are included in the article/Supplementary Material, further inquiries can be directed to the corresponding authors.
