# Supplementary material for: Enhancing the implantation of mechanical circulatory support devices using computational simulations
Source: Front Bioeng Biotechnol. 2024 Apr 25;12:1279268. doi: 10.3389/fbioe.2024.1279268 (PMC11084291; doi:10.3389/fbioe.2024.1279268)
Supplement: Supplementary file 1 [file DataSheet1.pdf]

Supplementary Material

1 The Three-Element Windkessel Model

For the outlets, the three-element Windkessel model RCR is used and the 0D – 3D coupling is a combination of medical parameters such as the compliance, aortic valve profile, and peripheral and distal resistance (Westerhof et al., 2019).

The three-element WK model can be represented by adding Ordinary Differential Equations (ODE) in the field functions (Siemens Community, 2020).

$$Q\left(1-\frac{R_p}{R_d}\right)+CR_p\frac{dQ}{dt}=\frac{P-P_{out}}{R_d}+C\frac{dP}{dt}$$

The flow rate and the pressure at the flow boundary are denoted by Q and P. The peripheral and distal resistances are represented by R_p and R_d, C is the compliance of the vessel, and P_{out} is the pressure at the exit of the Windkessel model, specified initially as zero. The variables are defined in global parameters.

The original equation can be rearranged, the pressure time derivative term can be approximated as a forward Euler finite difference, and the flow rate time derivative can be estimated as a backward difference using surface average reports and field history monitors for the volume flow rate and pressure.

$$P_{t+1}=P_t+\frac{\Delta t}{C}\left[Q\left(1+\frac{R_p}{R_d}\right)+CR_p\frac{Q_t-Q_{t-1}}{\Delta t}+\frac{P_{out}-P_t}{R_d}\right]$$

The solution is first order accurate and can be used to determine the pressure at the outlet of each artery.

The values RCR can be calculated relating the haemodynamic parameters to a circuit in parallel (Supplementary Figure 1) where the proximal resistance is related to a viscosity resistance, the distal resistance is related to the resistance of capillaries and veins, and the capacitor is equivalent to the vessel compliance.

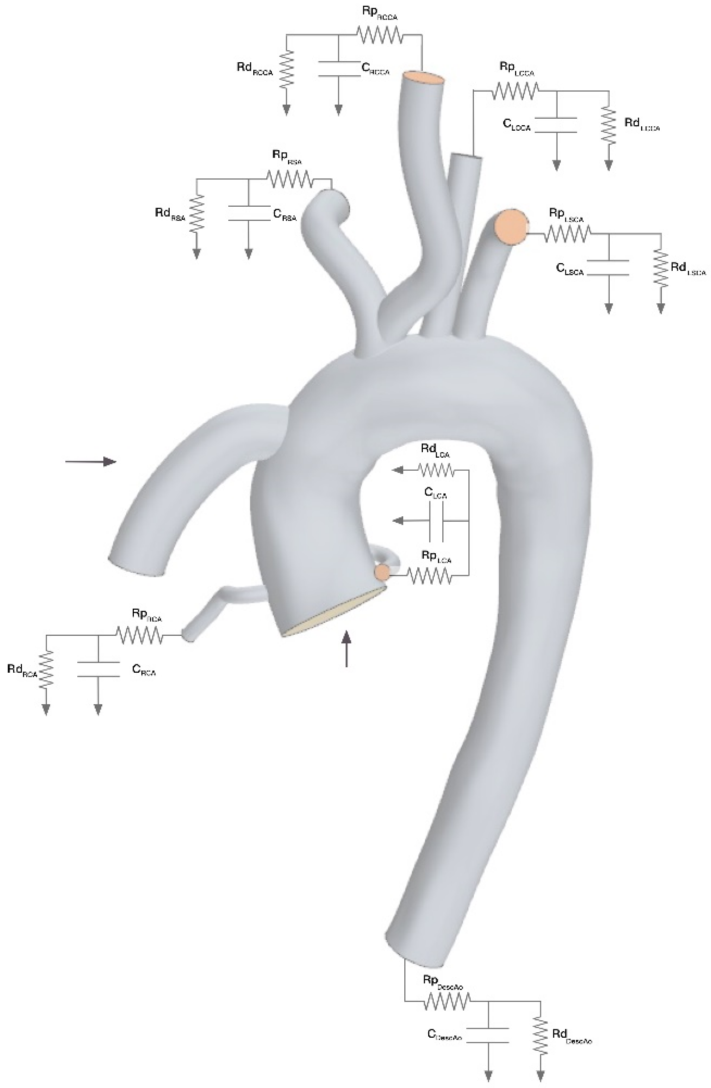

Supplementary Figure 1. RCR Windkessel aortic parallel circuit.

In a parallel circuit, the following relations can be applied to the aorta and its arteries.

$$\frac{1}{R_{Total}} = \frac{1}{R_{RCA_T}} + \frac{1}{R_{LCA_T}} + \frac{1}{R_{RSA_T}} + \frac{1}{R_{RCCA_T}} + \frac{1}{R_{LCCA_T}} + \frac{1}{R_{LSCA_T}} + \frac{1}{R_{DescAo_T}}$$

$$C_{Total} = C_{RCA} + C_{LCA} + C_{RSA} + C_{RCCA} + C_{LCCA} + C_{LSCA} + C_{DescAo}$$

$$R_{Total} = R_p + R_d$$

1.1 Total Resistance or Systemic Vascular Resistance

The cardiac output (CO) is the volume of blood pumped by each ventricle in 1 minute and is defined by the product of the Stroke Volume (SV), the volume of blood ejected from the ventricle with each beat, and the Heart Rate (HR), the number of times the heart beats per minute.

$$CO = SV * HR$$

Mean arterial pressure (MAP) is the average pressure in the arteries. This value is closer to value for diastolic pressure than systolic pressure because diastole lasts much longer than systole (Brzezinski, 1990).

$$MAP = \frac{2}{3} P_{diastolic} + \frac{1}{3} P_{systolic}$$

The pulse pressure (PP) is the difference between the systolic and the diastolic pressures. It represents the pressure increase in the vessels created by ventricular contraction. Pulse pressure declines to virtually zero by the time it reaches the capillaries.

$$PP = P_{systolic} - P_{diastolic}$$

The total resistance called the systemic vascular resistance (SVR) is calculated as follows:

$$R_{Total} = \frac{MAP - CVP}{CO}$$

where the Central Venous Pressure (CVP) is usually neglected in this formula (Brzezinski, 1990).

1.2 Outlets Resistance

There are two methods to estimate the outlet resistance: through the aortic 3D model and using percentages of flow from clinical documentation (Infantino, 2020).

The aortic 3D model is used to estimate the areas of the boundary outlets assuming that the outlets of higher area will have less resistance to the blood flow than the smaller outlets.

$$R_{Outlet} = \frac{\sum A_{Outlet}}{A_{Outlet}} R_{Total}$$

R_{Outlet}: Boundary outlet resistance

$\sum A_{Outlet}$: Summatory of the areas of all the boundary outlets

A_{Outlet}: Area of the boundary outlet of interest

1.3 Peripheral Resistance

The pulse wave velocity (PWV) is used as an indicator of arterial distensibility, which is the ability of the arteries to expand and contract with the cardiac pulsation. Patients' normotensives and hypertensives with a similar mean age recorded their PWV with a continuous doppler unit coupled with an electrocardiogram and found that the carotid-femoral PWV is positively correlated to MAP (O'Rourke, 1995). The peripheral resistance in the boundary outlet is calculated in function of PWV and the density of the blood, ρ.

$$R_{p_{Outlet}} = \frac{\rho PWV}{A_{Outlet}}$$

1.4 Total Compliance

The aortic compliance can be calculated in function of SV and the PP or using the graph of pressure and the decay time of diastolic aortic pressure, τ (Westerhof et al., 2019).

$$C_{Total} = \frac{SV}{PP}$$

$$C_{Total} = -\frac{\tau}{ln\left(\frac{P_{diastolic}}{P_{end-systole}}\right) R_{Total}}$$

1.5 Outlets Compliance

Assuming that the arteries outlets of higher area will have more compliance to the blood flow:

$$C_{Outlet} = \frac{A_{Outlet}}{\sum A_{Outlet}} C_{Total}$$

2 Turbulence Modelling

2.1 Wall Treatment for the Reynolds-Averaged Turbulence Models

A wall treatment is a set of near-wall modelling presumptions for calculating the effect of the turbulent velocity fluctuations on the averaged flow. Star CCM+ provides different wall treatments, depending on the turbulence model (Supplementary Figure 2). All-y^+ and two-layer all-y^+ models were used as wall treatment of the turbulence model on the transitional flow. The two-layer all-y^+ is recommended in flows where boundary layers play an important role. This applies to the aorta where the boundary layer is very thin and the vessel wall is moving radially (Febina et al., 2018).

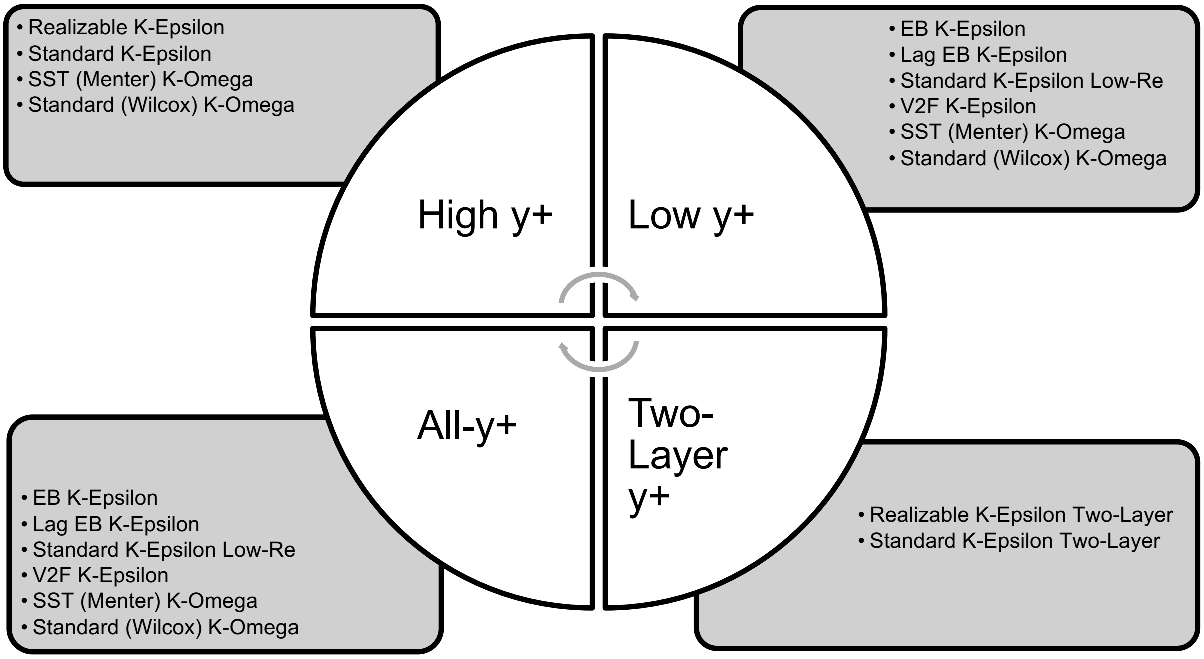

Supplementary Figure 2. Wall treatments available in Star CCM+.

In the All-y+ wall treatment, the simulation mimics the low-y^+ wall treatment for fine meshes and the high-y+ wall treatment for coarse meshes, while the all-y* wall treatment employs combined wall functions. This wall treatment is appropriate for a variety of near-wall mesh densities, including meshes with intermediate resolution. The intermediate resolution mesh occurs when the wall-cell centroid is located within the boundary layer's buffer zone $1>y^+>30$ (Siemens Digital Industries Software, n.d.).

The two-layer all-y^+ wall treatment follows the same methodology as the all-y^+ wall treatment and is available for two-layer turbulence models. To make it compatible with the two-layer formulation of the underlying turbulence model, particular values of the turbulence dissipation rate are enforced at the centroids of the near-wall cells. The two-layer all-y^+ wall treatment is only available with the two-layer $\kappa - \epsilon$ turbulence model (Siemens Digital Industries Software, n.d.). Supplementary Table 1 displays the wall treatment used in each turbulent model. All-y+ and the two-layer all-y^+ were used due to the complex geometry of the aorta and velocity scales associated with the model.

Supplementary Table 1. Wall treatment for the different turbulent models.

**Reynolds-Averaged Turbulence**	**Turbulence Model**	**Wall Treatment**
$\kappa - \epsilon$ model	Realizable $\kappa - \epsilon$ Two-Layer	Two-Layer All-y+
$\kappa - \omega$ model	Standard (Wilcox) $\kappa - \omega$	All-y+
$\kappa - \omega$ model	SST (Menter) $\kappa - \omega$	All-y+

2.2 Mesh Validation

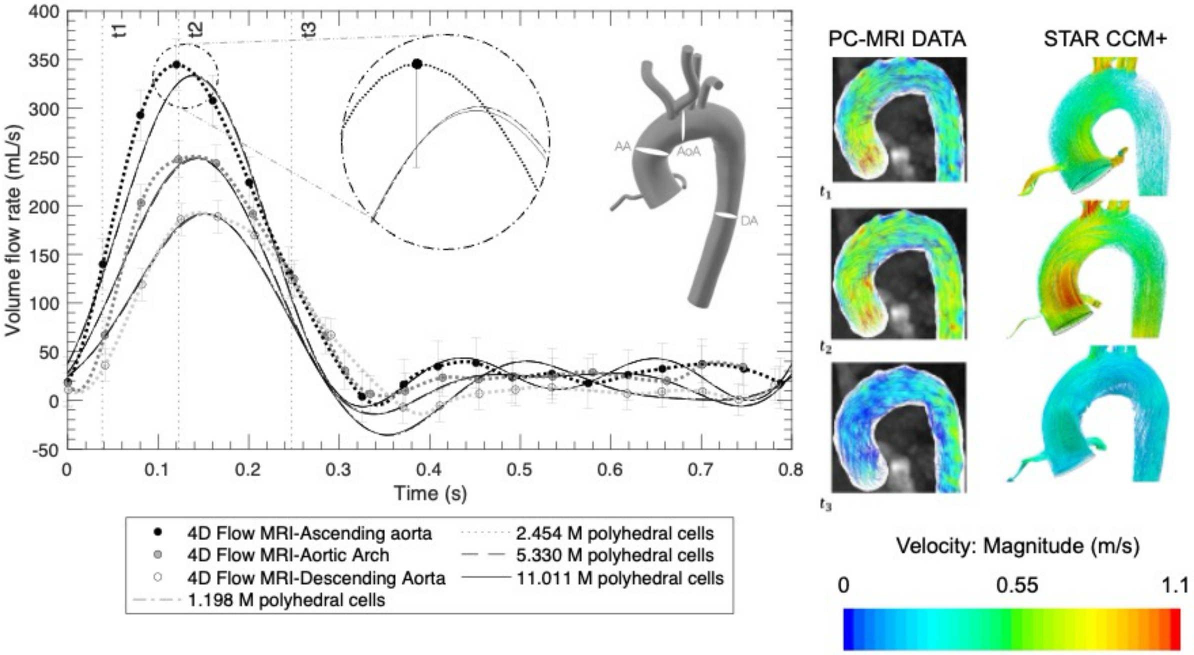

Supplementary Figure 3. Volume flow rate comparison with 4D flow data.

Table 1 shows a close agreement, considering that the 4D flow estimated values already have a margin of error introduced by the intra-voxel velocity distribution and partial volume effects as well as noise and data manipulation (Rothenberger et al., 2022).

Supplementary Table 2. Averaged volume flow rate comparison of unstructured mesh in different aortic sections.

	Ascending aorta		Aortic arch		Descending aorta	
	m L s^{-1}	%Error	m L s^{-1}	%Error	m L s^{-1}	%Error
Cardiac 4D flow MRI	90.10	*	70.92	*	49.58	*
1.2 M polyhedral cells	85.77	4.80	62.19	12.31	45.91	7.40
2.5 M polyhedral cells	85.78	4.79	62.12	12.40	45.93	7.36
5.3 M polyhedral cells	85.74	4.83	62.26	12.21	45.98	7.26
11 M polyhedral cells	85.70	4.88	62.18	12.32	45.97	7.28

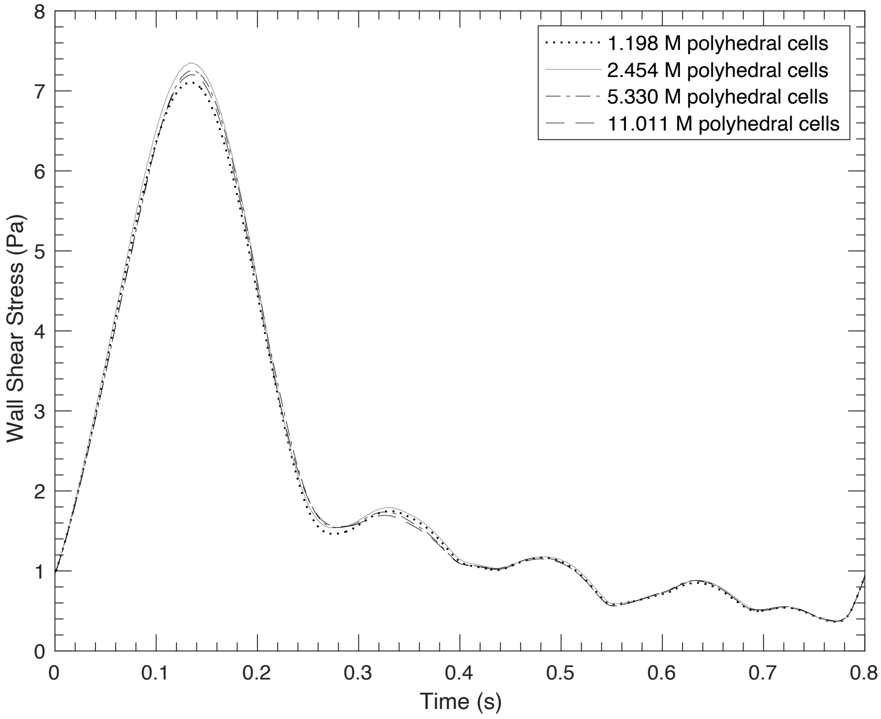

Supplementary Figure 4. Wall shear stress comparison with the different meshes.

There is a slight variation in the quantities of wall shear stress as, while all the curves follow the same tendency, changes are observed at the peak systolic for the maximum values of WSS where a convergence study is executed.

2.3 Turbulent Model Comparison Results

The two-equations turbulence models were simulated: realizable k−ε two-layer, Standard (Wilcox) k−ω, and SST (Menter) k−ω with its respective wall treatment. The results of the simulation are compared with the averaged volume flow rate values obtained from the 4D flow in three different aortic sections (Supplementary Figure 3).

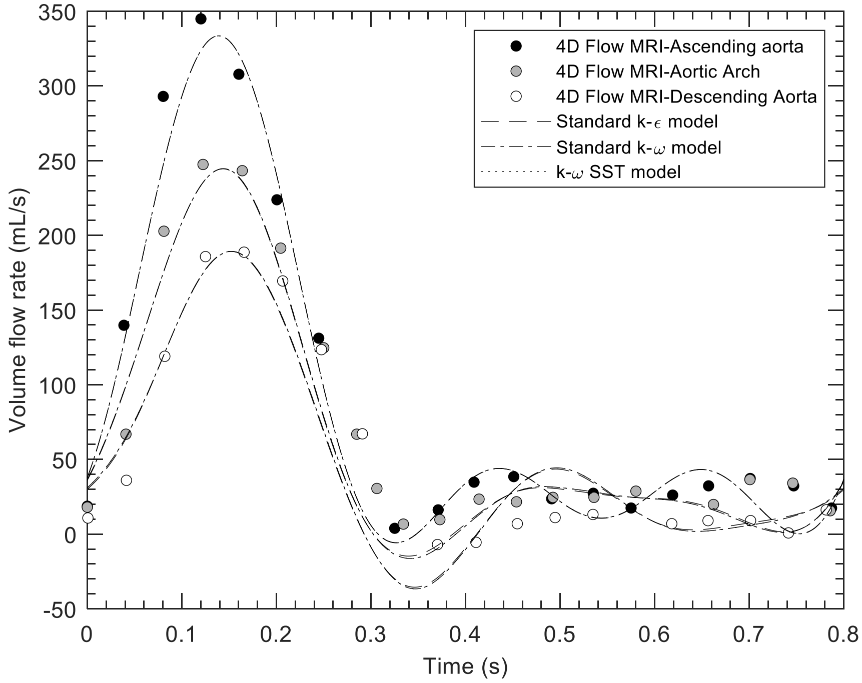

Supplementary Figure 5. Turbulence model simulation results comparison with the volume 4D flow rate.

For the present simulation, there is no significant difference between the turbulence models when using the wall treatment option from Star CCM+ (Supplementary Table 2), although the model that aligns slightly better with the data is the standard $\kappa - \epsilon$ model. The same comparison is executed with the normalised WSS (Supplementary Figure 4) and normalised pressure (Supplementary Figure 5).

Supplementary Table 3. Averaged volume flow rate comparison of different turbulent models.

	Ascending aorta		Aortic arch		Descending aorta	
	mL/s	Percentage Error	mL/s	Percentage Error	mL/s	Percentage Error
Cardia 4D flow MRI	90.10	*	70.92	*	49.58	*
Realizable $\kappa - \epsilon$ Two-Layer	85.73	4.85%	62.33	12.11%	46.38	6.45%
Standard (Wilcox) $\kappa - \omega$	85.72	4.86%	62.29	12.16%	46.37	6.47%
SST (Menter) $\kappa - \omega$	85.72	4.86%	62.29	12.16%	46.37	6.47%

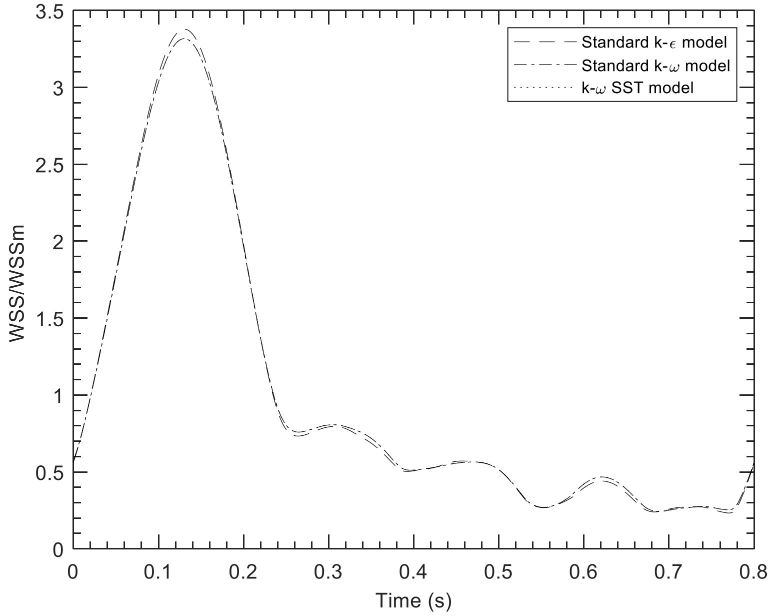

Supplementary Figure 6. Turbulence model comparison and normalized WSS.

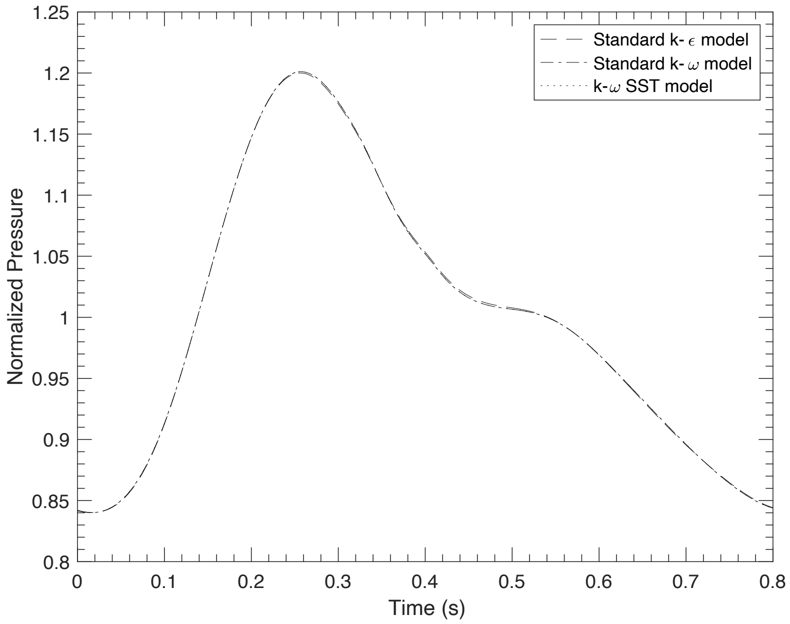

Supplementary Figure 7. Turbulence model comparison and normalized pressure.

References

Brzezinski, W. A. (1990). "Blood pressure," in *Clinical Methods: The History, Physical, and Laboratory Examinations*, eds. H. K. Walker, W. D. Hall, and J. W. Hurst (Boston: Butterworths). http://www.ncbi.nlm.nih.gov/books/NBK268/ [Accessed March 31, 2022].

Febina, J., Sikkandar, M. Y., and Sudharsan, N. M. (2018). Wall shear stress estimation of thoracic aortic aneurysm using computational fluid dynamics. *Comput. Math. Methods Med.* 2018, 7126532. doi: 10.1155/2018/7126532

Infantino, G. (2020). Reduced order methods for hemodynamics modelling. [PhD dissertation]. [Turin: The Polytechnic University of Turin] https://webthesis.biblio.polito.it/14783/ [Accessed March 31, 2022].

O'Rourke, M. (1995). Mechanical principles in arterial disease. *J. Hypertens.* 26, 2–9. doi: 10.1161/01.HYP.26.1.2

Siemens Community (2020). Windkessel model implementation. https://community.sw.siemens.com/s/article/Windkessel-Model-Implementation [Accessed March 31, 2022].

Siemens Digital Industries Software (n.d.). Simcenter STAR-CCM+ user guide, version 2021.2. https://docs.sw.siemens.com/documentation/external/PL20200805113346338/en-US/userManual/userguide/html/index.html#page/STARCCMP%2FGUID-2E8C2999-B5FC-4C95-B934-734059C9B045.html%23 [Accessed March 31, 2022].

Westerhof, N., Stergiopulos, N., Noble, M. I. M., and Westerhof, B. E. (2019). "The arterial Windkessel," in *Snapshots of Hemodynamics: An Aid for Clinical Research and Graduate Education*, eds. N. Westerhof, N. Stergiopulos, M. I. M. Noble, and B. E. Westerhof (Cham: Springer International Publishing), 207–216. doi: 10.1007/978-3-319-91932-4_25
